# Supplementary figures and images for: Broad Substrate-Specific Phosphorylation Events Are Associated With the Initial Stage of Plant Cell Wall Recognition in Neurospora crassa
Source: Front Microbiol. 2019 Nov 1;10:2317. doi: 10.3389/fmicb.2019.02317 (PMC6838226; doi:10.3389/fmicb.2019.02317)

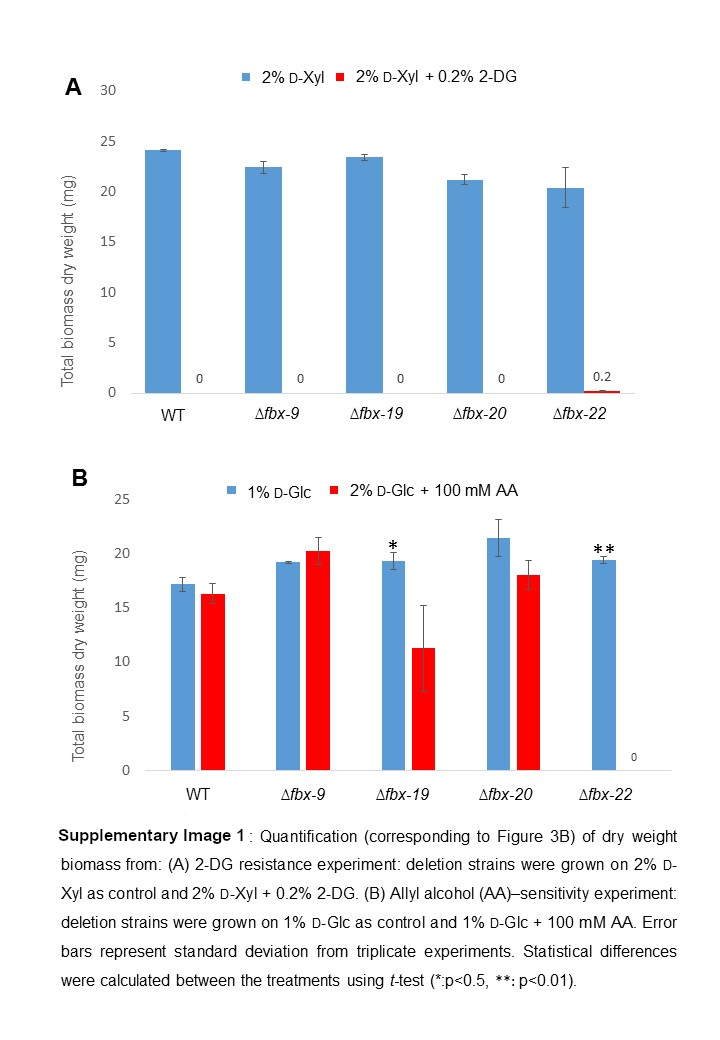

Supplement: Supplementary file 5 [file Image_1.jpg]

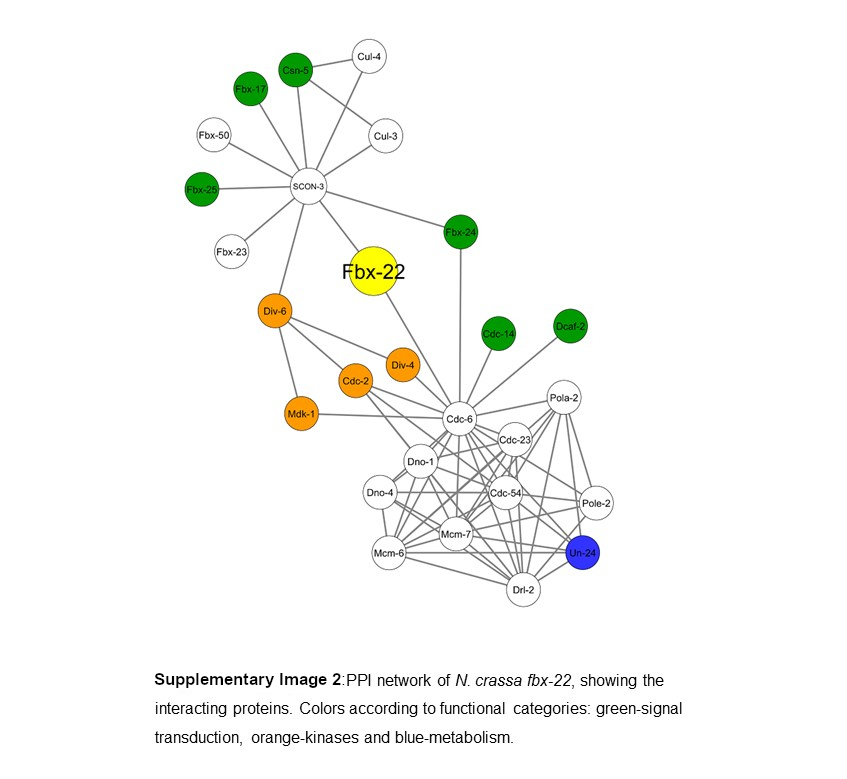

Supplement: Supplementary file 6 [file Image_2.jpg]
